# Anticipation of novelty recruits reward system and hippocampus while promoting recollection

**DOI:** 10.1016/j.neuroimage.2007.06.038

**Published:** 2007-10-15

**Authors:** Bianca C. Wittmann, Nico Bunzeck, Raymond J. Dolan, Emrah Düzel

**Affiliations:** a Wellcome Trust Centre for Neuroimaging, Institute of Neurology, University College London, 12 Queen Square, London WC1N 3BG, UK; b Institute of Cognitive Neuroscience and Department of Psychology, University College London, 17 Queen Square, London WC1N 3AR, UK; c Department of Neurology II and Centre for Advanced Imaging, Otto von Guericke University, Leipziger Str. 44, 39120 Magdeburg, Germany

## Abstract

The dopaminergic midbrain, which comprises the substantia nigra and ventral tegmental area (SN/VTA), plays a central role in reward processing. This region is also activated by novel stimuli, raising the possibility that novelty and reward have shared functional properties. It is currently unclear whether functional aspects of reward processing in the SN/VTA, namely, activation by unexpected rewards and cues that predict reward, also characterize novelty processing. To address this question, we conducted an fMRI experiment during which subjects viewed symbolic cues that predicted either novel or familiar images of scenes with 75% validity. We show that SN/VTA was activated by cues predicting novel images as well as by unexpected novel images that followed familiarity-predictive cues, an ‘unexpected novelty’ response. The hippocampus, a region implicated in detecting and encoding novel stimuli, showed an anticipatory novelty response but differed from the response profile of SN/VTA in responding at outcome to expected and ‘unexpected’ novelty. In a behavioral extension of the experiment, recollection increased relative to familiarity when comparing delayed recognition memory for anticipated novel stimuli with unexpected novel stimuli. These data reveal commonalities in SN/VTA responses to anticipating reward and anticipating novel stimuli. We suggest that this anticipatory response codes a motivational exploratory novelty signal that, together with anticipatory activation of the hippocampus, leads to enhanced encoding of novel events. In more general terms, the data suggest that dopaminergic processing of novelty might be important in driving exploration of new environments.

## Introduction

Single-neuron recordings in animals and recent functional magnetic resonance imaging (fMRI) studies in humans provide convergent evidence that the SN/VTA midbrain region is activated not only by reward (Schultz, 1998) but also by novel stimuli even in the absence of reinforcement (Schultz et al., 1997; Schott et al., 2004; Bunzeck and Duzel, 2006). SN/VTA activation by novelty raises the possibility that novelty might have intrinsic rewarding properties. If so, characteristics of reward processing, such as the temporal shift of responses in conditioning, should also hold for novelty processing. In reward anticipation paradigms, dopaminergic neurons code reward prediction when the contingency between a predictive stimulus and subsequent reward delivery has been learned. Specifically, these neurons respond to the first reliable predictor of reward but no longer to receipt of reward (Ljungberg et al., 1992; Schultz et al., 1992, 1997; Schultz, 1998). Whether novelty processing in the SN/VTA also shows these reward-related properties is unclear.

The hippocampus is critical in formation of episodic long-term memories for novel events (Vargha-Khadem et al., 1997; Duzel et al., 2001) and believed to provide the major input for a novelty signal in SN/VTA (Lisman and Grace, 2005). Dopamine released by SN/VTA neurons, in turn, is critical for stabilizing and maintaining long-term potentiation (LTP) and long-term depression (LTD) in hippocampal region CA1 (Frey et al., 1990, 1991; Huang and Kandel, 1995; Sajikumar and Frey, 2004; Lemon and Manahan-Vaughan, 2006; for a review see Jay, 2003). fMRI data have shown that joint SN/VTA and hippocampal activation is associated with successful long-term memory formation (Schott et al., 2006) and reward-related improvement in novel stimulus encoding (Wittmann et al., 2005; Adcock et al., 2006). In light of such converging evidence, recent models of hippocampus-dependent memory formation emphasize a functional relationship between novelty detection in the hippocampus and enhancement of hippocampal plasticity by novelty-induced dopaminergic modulation arising from the SN/VTA (Lisman and Grace, 2005). Therefore, the question whether the SN/VTA is activated by anticipating novelty goes beyond a conceptual understanding of the relationship between novelty and reward to embrace mechanisms of hippocampal plasticity. Furthermore, it has recently been suggested that understanding the relationship between novelty and reward-processing in SN/VTA might reveal links between motivation, novelty-seeking behavior and exploration (Bunzeck and Duzel, 2006; Knutson and Cooper, 2005).

**Fig. 1:**
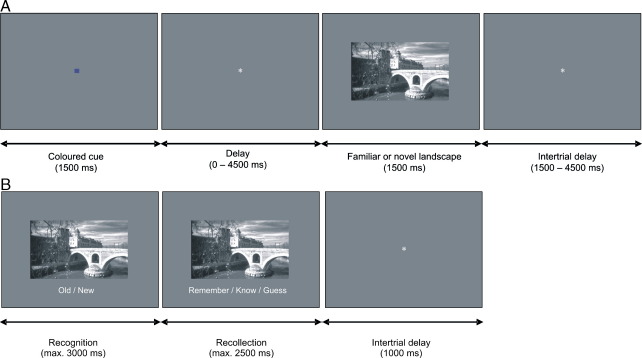
Experimental design. (A) Trial sequence for the study phase. After a familiarization phase, colored cues predicted with an accuracy of 75% whether a familiar or new picture followed. Participants were informed about the probabilities and asked to indicate by button press for each picture whether it was familiar or new. (B) Trial sequence for the memory test. Pictures that had been presented in the study phase one day earlier were shown randomly alternating with new distractor pictures. Participants first made an old/new decision, then reported the quality of their recognition memory according to the remember/know/guess procedure.

We investigated anticipatory responses to novel and familiar stimuli in an fMRI paradigm modeled upon reward anticipation procedures (Fig. 1). Colored squares served as cues that predicted subsequent presentation of novel or previously familiarized images of scenes. Subjects were instructed to attend to each cue and then indicate as quickly and accurately as possible whether the subsequent image was familiar or new. As the fMRI experiment required a large number of trials, we also conducted a purely behavioral version in which trial numbers were more optimal to assess how episodic memory performance was affected by anticipation of novelty using a remember/know paradigm (Tulving, 1985).

## Experimental procedures

### Subjects

Fifteen healthy adults (mean age [± SD] 24.5 ± 4.0 years, all right-handed, 7 male) participated in the experiment. All participants gave written informed consent to participate, and the study was in accordance with the guidelines of the ethics committee of the University of Magdeburg, Faculty of Medicine.

### Experimental paradigm

We used 245 greyscale landscape photographs with normalized luminance. Participants received written instructions including print-outs of five pictures that had been selected for familiarization. Before entering the scanner, each of these pictures was presented eight times on a computer screen in randomized order (duration: 1500 ms, ISI: 1200 ms) while participants were instructed to watch attentively. In the scanner, both anatomical and functional images were collected. Participants engaged in 12 sessions of 5.7 min duration, each containing 40 trials of 4.5–12 s length. During each trial, participants saw a yellow or blue square (1500 ms) indicating with 75% accuracy whether the following picture would be familiar or novel (see Fig. 1A for task and instructions). After a variable delay (0–4.5 s), a picture from the predicted category was shown in 75% of the trials, and a picture from the unpredicted category, novel following a familiarity cue and familiar following a novelty cue, was shown in 25% of the trials (1500 ms). Both categories were shown equally often. Participants indicated with a fast button press (right or left index or middle finger) whether the picture was from the familiar category or not. A fixation phase of variable duration followed (1.5–4.5 s). The cue colors associated with each picture category were counterbalanced across participants, as well as the responding hand and the assignment of the fingers to the categories.

### fMRI procedures

We acquired 226 echo-planar images (EPI) per session on a 3 T scanner (Siemens Magnetom Trio, Erlangen, Germany) with a TR of 1.5 s and a TE of 30 ms. Images consisted of 24 slices along the longitudinal axis of the midbrain (64 × 64 matrix; field of view: 19.2 cm; voxel size: 3 × 3 × 3 mm) collected in an interleaved sequence. This partial volume covered hippocampus, amygdala, brainstem (including diencephalon, mesencephalon, pons, and medulla oblongata) and parts of the prefrontal cortex. Scanner noise was reduced with ear plugs and subjects' head movements were minimized with foam pads. Stimulus sequence and timing were optimized for efficiency regarding reliable separation of cue- and outcome-related hemodynamic responses (Hinrichs et al., 2000). An inversion recovery EPI sequence (IREPI) was acquired for each subject to improve normalization. Scanning parameters were the same as for the EPI sequence but with full brain coverage.

Preprocessing and data analysis were performed using Statistical Parametric Mapping software implemented in Matlab (SPM2; Wellcome Trust Centre for Neuroimaging, Institute of Neurology, London, UK). EPI images were corrected for slice timing and motion and then spatially normalized to the Montreal Neurological Institute template by warping the subject's anatomical IREPI to the SPM template and applying these parameters to the functional images, transforming them into 2 × 2 × 2 mm sized voxels. They were then smoothed using a 4 mm Gaussian kernel.

For statistical analysis, the data were scaled voxel-by-voxel onto their global mean and high-pass filtered. Trial-related activity for each subject was assessed by convolving a vector of trial onsets with a canonical hemodynamic response function and its temporal derivatives (Friston et al., 1998). A general linear model (GLM) was specified for each participant to model effects of interest using two onsets per trial, one for cue onset and one for outcome onset (covariates were: novelty cue, familiarity cue, expected/unexpected novel outcome, expected/unexpected familiar outcome) and six covariates of no interest capturing residual motion-related artifacts. The following contrasts were analyzed: novel vs. familiar cues, novel vs. familiar outcomes, unexpected vs. expected outcomes, unexpected vs. expected novel outcomes and unexpected vs. expected familiar outcomes. After creating statistical parametric maps for each participant by applying linear contrasts to the parameter estimates, a second-level random effects analysis was performed to assess group effects. Given our a priori hypothesis of activation of the reward and hippocampal systems, the effects were tested for significance in one-sample *t*-tests at a threshold of *p* < 0.005, uncorrected, and a minimum cluster size of *k* = 5 voxels, unless otherwise stated. Spherical small volume correction was then carried out centered on the peak voxels, using diameters corresponding to the size of the structures [7.5 mm for activations in the anterior hippocampus (see Lupien et al., 2007) and 4.5 mm for activations in the substantia nigra (see Geng et al., 2006)]. Beta values of peak voxels in substantia nigra and hippocampus were extracted and corrected with the value of the HRF for general level of activation in the trial to yield percentage of signal change. All behavioral averages are given as mean values ± standard error of the mean (SEM).

To localize midbrain activity, activation maps were superimposed on a mean image of 33 spatially normalized magnetization transfer (MT) images acquired previously (Bunzeck and Duzel, 2006). On MT images, the substantia nigra can be easily distinguished from surrounding structures (Eckert et al., 2004). To assist the localization of activations, the peak voxels of each contrast were transferred to Talairach space (Talairach and Tournoux, 1988) using the Matlab function mni2tal.m (Matthew Brett, 1999) and matched to anatomical areas using the software Talairach Daemon Client (Lancaster et al., 2000; Version 1.1, Research Imaging Center, University of Texas Health Science Center at San Antonio). All stereotaxic coordinates are therefore given in Talairach space.

### Separate memory assessment

In a separate behavioral follow-up study motivated by the fMRI findings, 12 participants (2 male) completed the same familiarization and novelty anticipation procedures as implemented for the fMRI experiment. The behavioral experiment was separated from the fMRI experiment because the duration and number of stimuli in the fMRI were optimized to improve signal quality but too extensive to allow memory performance to remain above chance. Therefore, to facilitate memorization in the behavioral experiment, the number of trials containing expected novel pictures was reduced to 120, the number of unexpected novel pictures to 40. One day after the study session, participants completed a memory test containing all 160 novel pictures from the study phase (now ‘old’ pictures) and 80 new distractor pictures that the participants had not seen before (Fig. 1B). In this part of the study, participants made two consecutive decisions for each picture, both of which were cued by text presented below the picture. The first decision was to make an “old/new” judgement, the second decision was a “remember/know/guess” (after an “old” response), or a “sure/guess” (after a “new” response) judgement. Timing was self-paced, with a time limit for the decisions of 3 s and 2.5 s, respectively, followed by a 1 s fixation phase before presentation of the next picture.

## Results

### Behavioral results

For the study phase, a 2 × 2 × 2 ANOVA on participants' reaction times on correct trials with the factors picture category (novel/familiar), expectation (expected/unexpected) and group (scanned group/memory group) showed main effects of picture category and expectation and an interaction between group and picture category effect (see Table 1 for reaction times; category effect: *F*[1,25] = 31.57, *p* < 0.001; expectation effect: *F*[1,25] = 8.47, *p* < 0.01; interaction effect: *F*[1,25] = 5.49, *p* < 0.05). Post hoc paired *t*-tests confirmed that reaction times for both expected familiar pictures and expected novel pictures were significantly shorter than for the corresponding unexpected pictures (*p* < 0.01 and *p* < 0.05, respectively). Reaction times for both expected and unexpected familiar pictures were significantly shorter than for the corresponding novel pictures (*p* < 0.001 and *p* = 0.001, respectively). The interaction effect did not result from a significant category effect in only one participant group, as *t*-tests comparing reaction times to novel and familiar pictures were significant for both groups (*p* < 0.05 for the scanned group and *p* < 0.001 for the memory group). These results confirm that participants paid attention to the cues and used them to gain a behavioral advantage for the discrimination of novel and familiar pictures. Correct response rates did not differ between the categories or between groups (average for expected novel pictures: 95.1% ± 3.7%, for unexpected novel pictures: 94.1 ± 3.6%, for expected familiar pictures: 93.8% ± 3.9% and for unexpected familiar pictures: 93.4% ± 3.5%).

We then analyzed results from the memory test that was carried out 1 day after the study phase in the behavioral follow-up. A two-way ANOVA with the factors memory (corrected remember/know rates) and novelty anticipation (expected/unexpected) showed an interaction effect (*F*[1,11] = 5.66, *p* < 0.05). Post hoc paired *t*-test revealed a significantly higher difference between corrected remember/know rates for expected (8.9 ± 5%) than unexpected (0.9 ± 4%) novel pictures (*p* < 0.05; for response rates see Table 2). Further post hoc paired *t*-tests confirmed that neither corrected remember rate vs. corrected know rate nor expected vs. unexpected alone was significantly different. Proportion of guess responses did not differ between the categories (11.1 ± 2.3% for expected and 12.3 ± 2.4% for unexpected pictures).

We also analyzed the contributions of recollection and familiarity under an independence assumption on the basis of a widely accepted model (Yonelinas et al., 1996), according to which recollection represents a hippocampus-dependent threshold process whereas familiarity represents a signal-detection process that can be supported in the absence of an intact hippocampus. Recollection was estimated by subtracting the rate of remember false alarms (RFA) from the remember rate. Familiarity was estimated by first calculating familiarity responses (FR, see equation below) and then obtaining the corresponding d-prime value.

$$\mathrm{FR}=\frac{\left(\text{hitrate}-(\text{rem}-\mathrm{RFA})\right)}{1-(\text{rem}-\mathrm{RFA})}=\frac{\text{hitrate}-\mathrm{RE}}{1-\mathrm{RE}}$$

In order to be able to compare estimates of recollection (RE), which are response proportions in percent, and familiarity estimates (FE), which are *d*' values, both measures were transformed into *z*-scores before statistical analyses. A two-way ANOVA with the factors memory (recollection estimate/familiarity estimate) and novelty anticipation (expected/unexpected) confirmed the interaction effect obtained in the ANOVA on response rates (*F*[1,11] = 5.78, *p* < 0.05).

### fMRI results

**Fig. 2:**
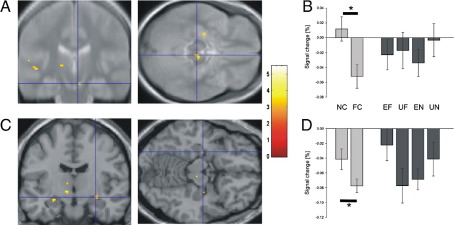
‘Novelty anticipation’ response: Hemodynamic activity for cues predicting novel pictures vs. cues predicting familiar pictures. (A) Cluster of activation in right SN/VTA. (B) Estimated percent signal change of the hemodynamic response for the two cues (light grey) and four outcome categories (dark grey). Talairach coordinates: [4, − 22, − 12]; error bars indicate SEM. (C) Clusters of activation in bilateral hippocampus. (D) Signal change for the two cues (light grey) and four outcome categories (dark grey). Talairach coordinates: [28, − 10, − 8]; error bars indicate SEM; (A, C) *p* < 0.005 (uncorrected); *p* < 0.05 (SVC); cluster size > 5 voxels. (B, D) NC—novelty cue, FC—familiarity cue, EF—expected familiar outcome, UF—unexpected familiar outcome, EN—expected novel outcome, UN—unexpected novel outcome. (B, D) Note that our experimental design did not allow efficient estimation of baseline activity, and thus the absolute values of the parameter estimates are poorly estimated (i.e. the value of 0 on the *y* axis is somewhat arbitrary), although the differences between parameters are well estimated (Josephs and Henson, 1999).

Cues leading to anticipation of novel pictures, in contrast to anticipation of familiar pictures, led to significantly higher activity in brain areas that form the dopaminergic system (left striatum; right midbrain, most likely the SN; Figs. 2A, B; Table 3), areas previously associated with reward anticipation (Knutson et al., 2001a,b; O'Doherty et al., 2002; for a review see Knutson and Cooper, 2005). For the outcome contrast, unexpected vs. expected novel outcomes also activated the right SN/VTA (Figs. 4A, B; Table 4). This activation pattern resembles an activation pattern seen in dopaminergic midbrain with reward paradigms where dopaminergic neurons report a prediction error in reward (Schultz et al., 1997). In contrast, activity in response to familiarity cues and unexpected vs. expected familiar pictures did not show this pattern. Thus, these results demonstrate parallels between the processing of novelty and reward in the SN/VTA.

**Fig. 3:**
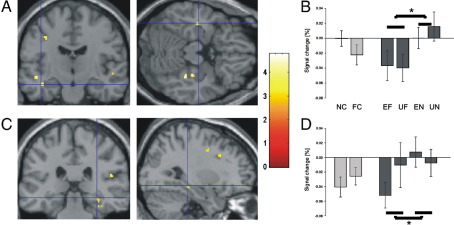
‘Novel outcome’ response: Hemodynamic activity for all novel pictures vs. all familiar pictures, independent of the preceding cue. (A) Cluster of activation in left hippocampus. (B) Estimated percent signal change of the hemodynamic response for the two cues (light grey) and four outcome categories (dark grey). Talairach coordinates: [− 40, − 14, − 14]; error bars indicate SEM. (C) Cluster of activation in right hippocampus. (D) Signal change for the two cues (light grey) and four outcome categories (dark grey). Talairach coordinates: [34, − 22, − 12]; error bars indicate SEM; (A, C) *p* < 0.005 (uncorrected); *p* < 0.05 (SVC); cluster size > 5 voxels. (B, D) NC—novelty cue, FC—familiarity cue, EF—expected familiar outcome, UF—unexpected familiar outcome, EN—expected novel outcome, UN—unexpected novel outcome. (B, D) Note that our experimental design did not allow efficient estimation of baseline activity, and thus the absolute values of the parameter estimates are poorly estimated (i.e. the value of 0 on the *y* axis is somewhat arbitrary), although the differences between parameters are well estimated (Josephs and Henson, 1999).

**Fig. 4:**
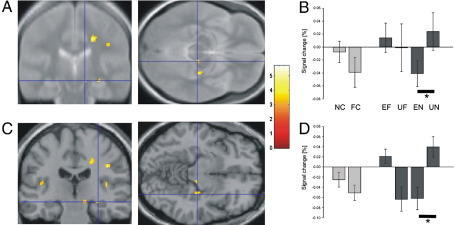
‘Unexpected novelty’ response: Hemodynamic activity for unpredicted novel pictures, i.e. novel pictures shown after cues predicting familiar pictures, vs. predicted novel pictures, i.e. novel pictures predicted by the preceding cue. (A) Cluster of activation in right SN/VTA. (B) Estimated percent signal change of the hemodynamic response for the two cues (light grey) and four outcome categories (dark grey). Talairach coordinates: [12, − 24, − 7]; error bars indicate SEM. (C) Cluster of activation in right hippocampus. (D) Signal change for the two cues (light grey) and four outcome categories (dark grey). Talairach coordinates: [30, − 22, − 7]; error bars indicate SEM; (A, C) *p* < 0.005 (uncorrected); *p* < 0.05 (SVC); cluster size > 5 voxels. (B, D) NC—novelty cue, FC—familiarity cue, EF—expected familiar outcome, UF—unexpected familiar outcome, EN—expected novel outcome, UN—unexpected novel outcome. (B, D) Note that our experimental design did not allow efficient estimation of baseline activity, and thus the absolute values of the parameter estimates are poorly estimated (i.e. the value of 0 on the *y* axis is somewhat arbitrary), although the differences between parameters are well estimated (Josephs and Henson, 1999).

In the hippocampus, both novelty anticipation and novel outcomes were associated with enhanced bilateral activity compared with anticipation and outcome of familiar stimuli (Figs. 2C, D and 3; Table 3). The right hippocampus was also more active for unexpected novel pictures than for expected novel pictures (Figs. 4C, D; Table 4). Furthermore, the left hippocampus (Talairach coordinates: − 36, − 14, − 14) showed higher activity for the presentation of all unexpected pictures in a contrast with all expected pictures, consistent with the hippocampal processing of contextual novelty (Ranganath and Rainer, 2003; Bunzeck and Duzel, 2006).

**Fig. 5:**
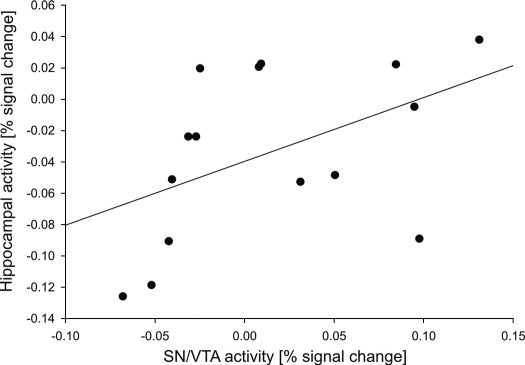
Correlation between SN/VTA activation and right hippocampal activity as tested on average percent signal change in response to novelty cues in the peak voxels of the ‘novelty vs. familiarity anticipation’ contrast.

In the cue phase, there was a significant positive correlation between right SN/VTA activation and right hippocampal activity as tested using average percent signal change in response to novelty cues in the peak voxels of the ‘novelty vs. familiarity anticipation’ contrast over participants (Pearson's *r* = 0.48, *p* < 0.05 one-tailed; Fig. 5). Thus, our data indicate a functional interaction as well as functional dissociations between the SN/VTA and the hippocampus in novelty processing.

## Discussion

Behaviorally, cue validity was associated with a significant effect on subjects' reaction times during discrimination of novel and familiar stimuli, showing that cues predicting novel or familiar events were processed by subjects. fMRI analysis revealed that cues predicting novel images elicited significantly higher SN/VTA activation than cues predicting familiar stimuli (Figs. 2A, B; Table 3). This SN/VTA activation pattern in response to novelty resembles a pattern found in reward paradigms where a response is seen to the earliest predictor of reward (Knutson et al., 2001a; Wittmann et al., 2005). Another property of reward processing in the SN/VTA, namely, increased activity for unexpected as compared to expected rewards (Schultz, 1998), was also paralleled by SN/VTA responses to novelty. SN/VTA activation was stronger in response to unexpected presentation compared with expected presentation of novel items (Figs. 4A, B; Table 4). Note that it is unlikely that anticipatory SN/VTA activation reflected contamination of the hemodynamic signal induced by subsequent novel stimuli as there was no SN/VTA activation by predicted novel stimuli or familiarity cues, demonstrating the effectiveness of the jittering protocol.

Our findings indicate that the similarity between novelty and reward goes beyond their common influence on SN/VTA-hippocampal circuits and raise the possibility that novelty itself is processed akin to a reward. This is compatible with a number of observations from animal research including data showing reduced self-administration of amphetamine during exploration of novel objects (Klebaur et al., 2001), the development of place preference for environments containing novel stimuli (Bevins and Bardo, 1999) and conditioning to novelty (Reed et al., 1996). However, this relationship between novelty and reward does not affect inferences derived from traditional reinforcement protocols, which work effectively with familiar stimuli. This speaks to the fact that in many situations it is clearly advantageous for an agent to form reward associations to highly familiar items. Nevertheless, our data do provide support for the idea that intrinsic reward properties of novel stimuli may underlie exploratory behaviors typically observed to novel contexts and items (Ennaceur and Delacour, 1988; Stansfield and Kirstein, 2006). Another property of SN/VTA neuronal coding of reward outcome is adaptive coding (Tobler et al., 2005), which is characterized by a different level of responding to the same expected reward value depending on the alternative rewards available in each context. Medium-value rewards lead to a higher dopaminergic response if presented in context with low-value rewards than in context with high-value rewards. This property of SN/VTA reward processing has not yet been replicated for novelty in humans. Indeed there is evidence that, unlike reward, novelty might not be coded adaptively in the human SN/VTA (Bunzeck and Duzel, 2006), suggesting functional differences between novelty and reward that bear further investigation.

The stimulus-related pattern of activity during novelty processing in the hippocampus differed from the pattern seen in the SN/VTA. Unlike SN/VTA, the hippocampus showed higher activity for expected novel stimuli themselves (Fig. 3). Moreover, the hippocampus was also more activated by contextual novelty (Lisman and Grace, 2005) independently of stimulus novelty, apparent in its response to the unpredicted presentation of familiar pictures. This confirms previous data (Bunzeck and Duzel, 2006), including findings indicating a sensitivity of this structure to mismatches within learned sequences (Kumaran and Maguire, 2006). The activation of the hippocampus by novel stimuli per se is well compatible with the so-called VTA-hippocampal loop model, according to which hippocampal novelty signals to the SN/VTA result from an intrahippocampal comparison of stimulus information with stored associations (Lisman and Grace, 2005). Hippocampal activation in response to novelty-predicting cues (Figs. 2C, D; Table 3), on the other hand, cannot be explained by this model. We suggest that a dopaminergic prediction signal induces hippocampal activation via dopaminergic input to CA1 (Jay, 2003), an interpretation compatible with a significant correlation between cue-related activity in SN/VTA and hippocampus found in this study.

Previous results indicate that several brain areas outside the mesolimbic system show differential anticipatory responses in reward paradigms. A recent example is the demonstration of such responses in primary visual cortex V1 (Shuler and Bear, 2006). These responses are hypothesized to be driven by dopaminergic modulation. A similar mechanism could apply to the processing of novelty. Irrespective of whether the dopaminergic midbrain drives the hippocampus or vice versa, coactivation of the hippocampus and SN/VTA could be associated with increased dopaminergic input to the hippocampus during anticipation. This, in turn, could induce a state that enhances learning for upcoming novel stimuli, a model that is computationally feasible (Blumenfeld et al., 2006).

In addition to the SN/VTA-hippocampal processing of novelty anticipation, there were also other brain regions showing activity in response to novelty cues, most notably areas in frontal cortex previously associated with novelty processing (Daffner et al., 2000; Table 3), and regions of the parahippocampal cortex (Duzel et al., 2003; Ranganath and Rainer, 2003). As our hypotheses were focused on SN/VTA and hippocampal processing, closer investigation of these results lies outside the scope of this study. Future investigation of the frontoparietal novelty network and its interactions with SN/VTA and hippocampus would add substantially to the growing understanding of novelty processing.

In keeping with the idea that preactivation of hippocampus during anticipation facilitates learning, our behavioral data show that expected novel pictures engendered a higher remember/know response difference than unexpected novel pictures when memory was tested 1 day later. A remember response requires recollection of context from the study episode and therefore reflects episodic memory in contrast to the familiarity-based, non-episodic aspect of recognition memory (Tulving, 1985; Duzel et al., 2001; Yonelinas et al., 2002). The hippocampus has been associated with successful episodic memory formation in previous studies (e.g. Brewer et al., 1998; Wittmann et al., 2005; Daselaar et al., 2006), and lesions of the hippocampus have been found to primarily impair the remember component of recognition (Duzel et al., 2001; Aggleton and Brown, 2006). We recently reported that memory for reward-predicting stimuli was also associated with a higher remember/know ratio as compared to stimuli that predicted the absence of reward (Wittmann et al., 2005), and this memory improvement was associated with increased SN/VTA and hippocampal activation in response to reward-predicting stimuli at the time of encoding. Our current results extend these findings to incorporate an SN/VTA-induced enhancement of hippocampal plasticity that is established by the earliest predictor of novelty. Interestingly, recent electrophysiological data from scalp recordings highlight a relationship between brain activity shortly preceding the onset of a new stimulus and episodic memory for that stimulus (Otten et al., 2006). Our data suggest that anticipation of novelty might be one mechanism through which prestimulus activity could modulate stimulus encoding. Our findings also extend recent fMRI data where reward expectancy and anticipation of an emotional stimulus were found to improve memory (Adcock et al., 2006; Mackiewicz et al., 2006).

The functional and anatomical overlap between reward and novelty processing in the SN/VTA might well serve to reinforce exploratory behavior, enabling animals to find new food sources and encode their location, thereby enhancing survival. An interesting avenue for future research will be to determine the relationship between novelty anticipation and a novelty-seeking personality trait. In humans, increased novelty-seeking is associated with gambling and addiction (Spinella, 2003; Hiroi and Agatsuma, 2005) raising the possibility of a trade-off between beneficial effects of anticipating novelty in memory and adverse effects in relation to addiction. A better understanding of the relationship between novelty anticipation, memory formation and novelty seeking could also inform research on the specific memory deficits found in dopaminergic dysfunction such as Parkinson's disease and schizophrenia.

In single-cell animal studies of reward processing, the observation that the SN/VTA responds to reward prediction as well as to unexpected reward has inspired ‘temporal difference’ (TD) models of reward processing (Schultz, 1998, 2002). It should be noted that, in our study, fMRI activations for novelty anticipation and unexpected novelty were located in slightly different portions within the SN/VTA. This raises the possibility that there might be regional response differences between reward prediction and unexpected reward responses in animals as well, and that single-neuron studies of novelty anticipation and unexpected novelty might also show that corresponding neuronal responses are located within different portions of the SN/VTA. A caveat here is the fact that we cannot exclude the possibility that in our study the same neuronal population that responded to novelty prediction also responded to unexpected novelty.

In summary, our fMRI data indicate that the hippocampal formation and the SN/VTA serve partly different functions in the prediction and processing of novelty. The SN/VTA processes predictability and the hippocampus the anticipated and actual presence of novelty in a given context. Together with our behavioral data, our findings suggest that the coactivation of SN/VTA and hippocampus to the earliest predictor of novelty in the prestimulus phase leads to an enhanced memory formation for the upcoming novel stimulus. These findings provide evidence for a close relationship between the processing of reward and stimulus novelty and extend recent models of dopaminergic–hippocampal interaction. They underline the importance of the prestimulus period for episodic encoding. Effects of novelty on encoding might therefore depend on induction of an anticipatory state in the medial temporal memory system, mediated by modulatory influences from dopaminergic midbrain areas. However, fMRI data do not provide direct evidence for the involvement of specific neurotransmitter systems. Notwithstanding, fMRI is a valuable tool to investigate event-related activity in the SN/VTA in humans. The integration of molecular genetic approaches into neuroimaging (Schott et al., 2006) and pharmacological fMRI might help to further elucidate the role of neuromodulatory transmitter systems in human novelty processing and the relationship between SN/VTA responses and dopaminergic neurotransmission.

## Figures and Tables

**Table 1:** Reaction times (in ms ± SEM) for correctly categorized pictures from the two picture categories (familiar/novel) and in relation to the preceding cue (expected/unexpected) for the two test groups

		Familiar pictures	Novel pictures
Scanned group	Expected	687 ± 32	723 ± 32
Unexpected	718 ± 26	746 ± 29
Memory group	Expected	602 ± 28	687 ± 31
Unexpected	642 ± 40	713 ± 34

**Table 2:** 

		Hits (%)	False alarms (%)	Corrected rate (%)
Remember	Expected	24.1 ± 5	4.2 ± 2	20.0 ± 12
Unexpected	19.4 ± 4	15.3 ± 10
Know	Expected	23.4 ± 2	12.2 ± 2	11.7 ± 8
Unexpected	26.6 ± 3	15.0 ± 7

**Table 3:** Novelty anticipation response: anatomical locations of regions active during anticipation of novel pictures vs. anticipation of familiar pictures

Area	Left/Right	Cluster size	Talairach coordinates			*T* value
*x*	*y*	*z*
Insula, BA 13	R	5	34	26	12	3.97
L	5	− 32	− 1	15	3.68
L	5	− 44	− 15	6	3.49
R	26	53	− 30	18	4.12
Middle frontal gyrus, BA 6	R	6	30	4	38	3.35
L	5	− 32	− 3	52	3.52
Precentral gyrus, BA 6	L	5	− 30	1	26	3.51
L	33	− 48	− 1	28	5.37
R	6	34	− 4	32	4.72
R	6	32	− 5	50	3.91
R	8	50	− 6	39	3.64
Precentral gyrus, BA 4	R	23	28	− 23	51	5.49
Cingulate gyrus, BA 23	L	12	− 2	− 14	28	4.85
Temporal gyrus, BA 42	L	9	− 61	− 15	8	3.6
Superior temporal gyrus, BA 22	R	16	55	− 17	1	4.01
Superior temporal gyrus, BA 41	L	10	− 50	− 23	5	4.17
R	6	42	− 41	6	3.6
Inferior parietal lobule, BA 40	R	7	48	− 31	31	4.19
Parahippocampal gyrus, BA 30	R	8	16	− 35	− 5	3.77
R	6	12	− 45	− 3	4.35
Parahippocampal gyrus, BA 36	L	12	− 18	− 36	− 18	4.67
R	8	4	− 45	− 4	3.7
Putamen	L	13	− 22	3	15	3.77
Thalamus	L	8	− 10	− 8	2	3.56
L	5	− 18	− 23	7	3.51
R	8	18	− 24	− 4	3.96
Hippocampus	R	11	28	− 10	− 8	5.17
L	5	− 26	− 12	− 11	3.56
Subthalamic nucleus	L	5	− 8	− 12	− 3	4.05
SN/VTA	R	10	4	− 22	− 12	4.28
Caudate	L	7	− 20	− 36	15	4.1

Data are thresholded at *p* < 0.005, uncorrected, and only clusters with > 5 voxels are reported.

**Table 4:** ‘Unexpected novelty’ response: anatomical locations of regions activated more strongly at outcome by unexpected novel pictures than by expected novel pictures

Area	Left/Right	Cluster size	Talairach coordinates			*T* value
*x*	*y*	*z*
Medial frontal gyrus, BA 9	L	6	− 38	15	34	3.52
Precentral gyrus, BA 6	L	5	− 38	− 4	30	3.58
Precentral gyrus, BA 4	R	43	46	− 8	43	4.64
Cingulate gyrus, BA 23	L	5	0	− 14	27	3.7
Postcentral gyrus, BA 2	R	10	42	− 20	32	5.05
Insula, BA 13	R	5	40	− 21	12	3.72
L	6	− 40	− 21	14	3.42
Cingulate gyrus, BA 31	R	31	20	− 23	40	5.7
Thalamus	L	10	− 12	− 8	2	4.21
Hippocampus	R	11	30	− 22	− 7	3.95
Substantia nigra	R	6	12	− 24	− 7	3.93
Caudate	L	5	− 22	− 34	16	3.54

Data are thresholded at *p* < 0.005, uncorrected, and only clusters with > 5 voxels are reported.
